# The Impact of Cognitive Load on the Spatial Deployment of Visual Attention: Testing the Role of Interhemispheric Balance With Biparietal Transcranial Direct Current Stimulation

**DOI:** 10.3389/fnins.2019.01391

**Published:** 2020-01-10

**Authors:** Rebecca E. Paladini, Fluri A. M. Wieland, Lien Naert, Mario Bonato, Urs P. Mosimann, Tobias Nef, René M. Müri, Thomas Nyffeler, Dario Cazzoli

**Affiliations:** ^1^Gerontechnology and Rehabilitation Group, University of Bern, Bern, Switzerland; ^2^Department of Psychology, University of Bern, Bern, Switzerland; ^3^Department of Experimental Psychology, Ghent University, Ghent, Belgium; ^4^Department of General Psychology, University of Padua, Padua, Italy; ^5^ARTORG Center for Biomedical Engineering Research, University of Bern, Bern, Switzerland; ^6^Perception and Eye Movement Laboratory, Departments of Neurology and BioMedical Research, Inselspital, Bern University Hospital, University of Bern, Bern, Switzerland; ^7^Neurocenter, Luzerner Kantonsspital, Lucerne, Switzerland

**Keywords:** visuospatial attention, non-spatial attention, cognitive load, transcranial direct current stimulation, bilateral tDCS

## Abstract

In healthy individuals, increasing cognitive load induces an asymmetric deployment of visuospatial attention, which favors the right visual space. To date, the neural mechanisms of this left/right attentional asymmetry are poorly understood. The aim of the present study was thus to investigate whether a left/right asymmetry under high cognitive load is due to a shift in the interhemispheric balance between the left and right posterior parietal cortices (PPCs), favoring the left PPC. To this end, healthy participants completed a visuospatial attention detection task under low and high cognitive load, whilst undergoing biparietal transcranial direct current stimulation (tDCS). Three different tDCS conditions were applied in a within-subjects design: sham, anodal left/cathodal right, and cathodal left/anodal right stimulation. The results revealed a left/right attentional asymmetry under high cognitive load in the sham condition. This asymmetry disappeared during cathodal left/anodal right tDCS, yet was not influenced by anodal left/cathodal right tDCS. There were no left/right asymmetries under low cognitive load in any of the conditions. Overall, these findings demonstrate that attentional asymmetries under high cognitive load can be modulated in a polarity-specific fashion by means of tDCS. They thus support the assumption that load-related asymmetries in visuospatial attention are influenced by interhemispheric balance mechanisms between the left and right PPCs.

## Introduction

The spatial allocation of visual attention can be affected by non-spatial attentional aspects, such as cognitive load. With increasing cognitive load, for instance in dual task paradigms (e.g., concurrently completing a non-spatial and a spatial attentional task), healthy individuals show a rightward shift in visuospatial attentional deployment (Pérez et al., 2009; Naert et al., 2018). This rightward shift may be influenced by the effect of cognitive load on the interhemispheric balance between the dorsal networks directing visual attention in space (Kinsbourne, 1993; Corbetta and Shulman, 2011). In particular, a higher cognitive load would favor activations within the left-hemispheric dorsal network relatively more than in the right-hemispheric dorsal network, thus triggering a rightward shift in attentional allocation (O’Connell et al., 2011). We aimed to test this hypothesis by applying bihemispheric transcranial direct current stimulation (tDCS) to simultaneously modulate the excitability of the left and the right posterior parietal cortices (PPCs), crucial nodes of the dorsal attention networks of the two hemispheres (Corbetta and Shulman, 2002), with reversed polarities. Such a bihemispheric electrode montage was shown to be particularly suitable to modulate the interhemispheric excitability balance between two brain regions (Sehm et al., 2012, 2013; Benwell et al., 2015; Li et al., 2015). We hypothesized that further shifting the interhemispheric balance in favor of the left PPC (excitatory anode over the left PPC, and inhibitory cathode over the right PPC; henceforth referred to as AL/CR) would exacerbate the rightward attentional shift under high cognitive load. In contrast, the reversed montage (inhibitory cathode over the left PPC, and excitatory anode over the right PPC; henceforth referred to as CL/AR) would contribute to restore interhemispheric balance, thereby reducing the rightward attentional shift under high cognitive load.

## Materials and Methods

Twenty-five healthy subjects (20 right-handed; eight men; mean age = 23.16 years, SD = 3.04), with no history of psychiatric or neurological disorders, volunteered in the study after giving written informed consent. The study was approved by the Ethics committee of the University of Bern, and was conducted according to the principles of the latest version of the Declaration of Helsinki.

**FIGURE 1:**
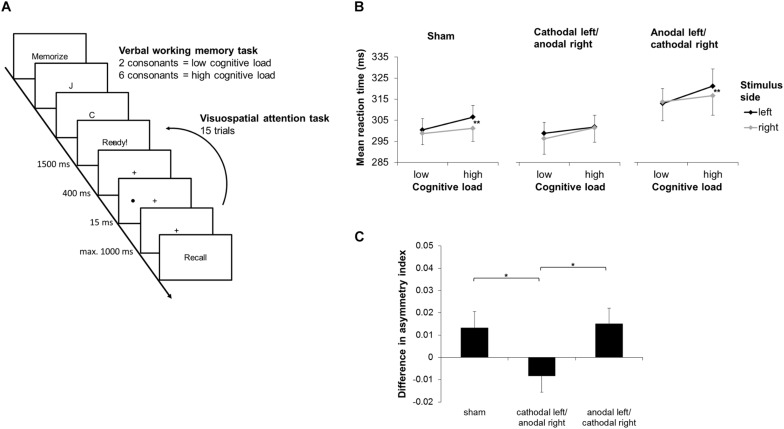
Design and results of the dual task paradigm with transcranial direct current stimulation (tDCS). **(A)** Exemplary depiction of a sub-block of the dual task. **(B)** Mean reaction times (RTs), according to stimulation condition, cognitive load, and stimulus side. *Post hoc* tests revealed no significant left-right asymmetries in any of the stimulation conditions during low cognitive load. However, during high cognitive load, significantly slower responses were observed for left- with respect to right-sided targets in the sham condition; this asymmetry disappeared in the cathodal left/anodal right (CL/AR) condition, and it remained unchanged in the anodal left/cathodal right (AL/CR) condition. Asterisks depict significant *post hoc* tests (^∗∗^*p* < 0.01). **(C)** Mean differences in the RT laterality index between high and low cognitive load, for each stimulation condition. Values >0 indicate a rightward attentional shift, whereas values <0 a leftward attentional shift. *Post hoc* tests revealed significant differences between the sham and the CL/AR tDCS conditions, as well as between the AL/CR and the CL/AR tDCS conditions, yet not between the sham and the AL/CR tDCS conditions. Asterisks depict significant *post hoc* tests (^∗^*p* < 0.05).

All participants took part in three sessions, each with a different biparietal tDCS protocol, in counterbalanced order across participants: (1) AL/CR; (2) CL/AR; (3) sham stimulation. A DC stimulator (neuroConn, Ilmenau, Germany) was used to apply tDCS. The two electrodes (5 × 7 cm; placed in sponges and wet with saline solution) were placed bihemispherically over the PPCs, i.e., P3 and P4 according to the 10–20 international electroencephalography (EEG) system. At the beginning of each session, participants completed a sleep quality questionnaire (Görtelmeyer, 2011; to assess how many hours they slept prior to the sessions), and a visual analog scale (VAS; to indicate their subjective level of alertness). The participants then completed a dual task (adapted from Naert et al., 2018; Figure 1A), encompassing a non-spatial verbal working memory task, which allowed to manipulate cognitive load, and a visuospatial attention detection task. In the non-spatial verbal working memory task, participants were asked to memorize 2 (low cognitive load) or 6 (high cognitive load) consonants. Whilst engaged in retaining the consonants, they performed the visuospatial attention detection task, i.e., react as quickly as possible upon appearance of a lateralized stimulus (i.e., a black dot, 0.86° visual angle, presented at 15° eccentricity), whilst maintaining central fixation. Each sub-block of the dual task contained one working memory trial and 15 subsequent detection trials (five left-sided, five right-sided, five catch trials without target; in randomized order). Overall, participants completed two blocks with low and two blocks with high cognitive load (in alternating sequence between low and high cognitive load; the order thereof, i.e., low cognitive load first or high cognitive load first, being counterbalanced over participants), each block containing five sub-blocks. Participants were asked to exclusively use their dominant hand for the detection task. tDCS (1.5 mA during 20 min; switched off after 30 s for sham stimulation) was applied throughout the dual task. Finally, after finishing the dual task, participants again completed the VAS.

Statistical analyses were conducted using SPSS (Version 23, IBM Statistics). In a first step, to ensure that participants’ alertness level did not differ between the sessions, a repeated-measures analysis of variance (ANOVA) with the within-subjects factor “stimulation condition” (levels: sham, CL/AR, AL/CR) was computed on the number of hours participants had slept prior to each session. Furthermore, to investigate participants’ subjective level of alertness between the sessions and pre/post the dual task, a repeated-measures ANOVA with the within-subjects factors “stimulation condition” (levels: sham, CL/AR, AL/CR) and “time point” (levels: pre, post dual task) was computed on the scores obtained from the VAS. In a second step, the reaction times (RT) in the visuospatial attention detection task were analyzed. For each participant, we calculated the mean reaction time according to stimulation condition, cognitive load condition, and stimulus presentation side. A repeated-measures ANOVA with the within-subjects factors “stimulation condition” (levels: sham, CL/AR, AL/CR), “cognitive load” (levels: low, high), and “stimulus side” (levels: left, right) was then computed. Subsequently, in order to further investigate the changes occurring between the two cognitive load conditions, we: (a) calculated a RT laterality index, according to the formula proposed by Newman et al. (2012): laterality index = (mean RT left-sided targets – mean RT right-sided targets)/(mean RT left-sided and right-sided targets); (b) we subsequently subtracted, within each stimulation condition, the laterality index for high minus the one for low cognitive load. A repeated-measures ANOVA with the within-subjects factor “stimulation condition” (levels: sham, CL/AR, AL/CR) was then calculated on the resulting data.

Since reaction time data are typically non-normally distributed, transformations are often applied to reduce deviations from normality (Marmolejo-Ramos et al., 2015; Vetter, 2017). Thereby, the square root transformation has been shown to be effective in several reaction time studies (e.g., Smoski et al., 2009; Sturz and Diemer, 2010). To ensure that our findings were not due to departures from normality in the reaction time data distribution, we thus applied a square root transformation and repeated the statistical analyses on the transformed data. All analyses yielded the same patterns of results as for the untransformed data. Thus, to avoid redundancies (as well as issues with the direct interpretation of transformed data; Whelan, 2008), in the results section we exclusively report the findings of the analyses on the untransformed data.

Concerning accuracy in the visuospatial attention detection test, we assessed correct responses according to stimulation condition, cognitive load condition, and trial type (i.e., left-sided targets, right-sided targets, or catch trials), by calculating the percentage of correct responses out of the 50 trials presented in each of the combinations of the three aforementioned factors. A repeated-measures ANOVA with the within-subjects factors “stimulation condition” (levels: sham, CL/AR, AL/CR), “cognitive load” (levels: low, high), and “trial type” (levels: left-sided targets, right-sided targets, catch trials) was then computed.

For all analyses, if the sphericity assumption was not met, the degrees of freedom (and thus the *P*-values) were corrected according to the Huynh-Feldt procedure. All *post hoc* tests were conducted by means of Duncan’s Multiple Range tests.

## Results

There were no significant differences in participants’ sleep duration prior to each stimulation condition [*F*(2,48) = 0.63, *p* = 0.537], nor in participants’ subjective level of alertness between and within (pre vs. post dual task) stimulation conditions [stimulation session: *F*(2,48) = 0.832, *p* = 0.442; time point: *F*(1,24) = 0.036, *p* = 0.851; stimulation session × time point: *F*(1.616,38.791) = 0.769, *p* = 0.445].

Regarding the dual task, the repeated-measures ANOVA on mean RTs yielded significant main effects of the factors “stimulation condition” [*F*(2,48) = 3.931, *p* = 0.026] and “cognitive load” [*F*(1,24) = 5.285, *p* = 0.031]. Concerning the former, *post hoc* tests revealed overall increased RTs in the AL/CR compared to the CL/AR and sham stimulation condition. There was no significant difference between sham stimulation and CL/AR. Regarding the main effect of “cognitive load,” RTs were significantly higher in the high compared to the low cognitive load condition. Critically, the ANOVA yielded a significant interaction “stimulation condition × cognitive load × stimulus side” [*F*(2,48) = 3.271, *p* = 0.047].

As revealed by *post hoc* tests, there were no significant left/right asymmetries in any of the stimulation conditions under low cognitive load. Yet, under high cognitive load, RTs were significantly higher for left- compared to right-sided targets in the sham condition. This asymmetry disappeared in the CL/AR condition, but it remained unchanged in the AL/CR condition. Significant *post hoc* results are depicted in Figure 1B. No other effects were significant [stimulus side: *F*(1,24) = 1.268, *p* = 0.271; stimulation condition × cognitive load: *F*(2,48) = 0.156, *p* = 0.856; stimulation condition × stimulus side: *F*(2,48) = 0.817, *p* = 0.448; cognitive load × stimulus side: *F*(1,24) = 2.253, *p* = 0.146].

Concerning the changes in the laterality index, the repeated-measures ANOVA revealed a significant main effect of the factor “stimulation condition” [*F*(2,48) = 3.427, *p* = 0.041; Figure 1C]. *Post hoc* tests revealed significant differences between the sham and the CL/AR tDCS conditions, as well as between the AL/CR and the CL/AR tDCS conditions, yet not between the sham and the AL/CR tDCS conditions. Hence, compared to the CL/AR tDCS condition, participants showed a significant rightward attentional shift in the AL/CR and the sham condition under high cognitive load.

Concerning accuracy in the visuospatial attention detection task, participants showed a very high rate of correct responses (overall accuracy of 98.698%). The repeated-measures ANOVA revealed a significant main effect of the factor “trial type” [*F*(2,48) = 4.81, *p* = 0.012]. Although *post hoc* tests failed to reach significance (all *p*’s > 0.05), participants seemed to be slightly more accurate in catch trials (i.e., correctly refraining from responding; mean = 99.16%, SD = 1.123) than in trials with left- or right-sided targets (mean = 98.373%, SD = 1.681; and, mean = 98.56%, SD = 1.536, respectively). No other effects were significant (stimulation condition: *F*(1.313,31.512) = 1.621, *p* = 0.216; cognitive load: *F*(1,24) = 1.852, *p* = 0.186; stimulation condition × cognitive load: *F*(2,48) = 1.494, *p* = 0.235; stimulation condition × trial type: *F*(4,96) = 0.781, *p* = 0.540; cognitive load × trial type: *F*(2,48) = 0.428, *p* = 0.654; stimulation condition × cognitive load × trial type: *F*(2.602,62.449) = 1.266, *p* = 0.293).

## Discussion

In the present study, we aimed to investigate the neural substrates of the rightward attentional shift observed under high cognitive load in healthy subjects, by directly modulating the interhemispheric interactions between the PPCs with biparietal tDCS. The results demonstrated: (a) a left/right asymmetry (shorter RTs for right- compared to left-sided targets) under high but not low cognitive load in the sham condition, and (b) polarity-specific effects of real stimulation, i.e., CL/AR tDCS canceled this asymmetry under high cognitive load, whereas AL/CR tDCS had no significant effect.

The rightward attentional shift under high cognitive load, as observed in the sham condition, is in line with previous findings in healthy individuals (Pérez et al., 2009; Naert et al., 2018). This phenomenon has been hypothesized to rely on a shift in the interhemispheric balance between the dorsal attentional networks, favoring the left hemisphere (O’Connell et al., 2011). Accordingly, correlational findings showed increased alpha-power (i.e., a decreased excitability) over the right relative to the left PPC under high cognitive load (Pérez et al., 2009). Our results provide strong support for this hypothesis, by means of a causal interference approach. The disappearance of the load-related left/right attentional asymmetry during CL/AR tDCS can be interpreted as a rebalancing of the interhemispheric activation imbalance between the two PPCs. This effect is not only specific (i.e., not observable during sham stimulation), but also polarity-specific (i.e., not observable during AL/CR tDCS).

These results also fit well with findings in neglect patients, who show a rightward shift in spatial attentional allocation. This rightward shift is due to an interhemispheric imbalance, with a relatively hypoactive right and a relatively hyperactive left dorsal attention network (Corbetta and Shulman, 2011), and is exacerbated under high cognitive load (Sarri et al., 2009; Bonato, 2012). Yet, inhibitory transcranial magnetic stimulation (TMS) over the left PPC has been shown to significantly reduce the detrimental effects of high cognitive load (Cazzoli et al., 2015).

Interestingly, AL/CR tDCS did not exacerbate the rightward attentional shift under high cognitive load. Previous studies found that pre-existing attentional biases can be reversed, yet not exacerbated, by means of tDCS stimulation (biparietal and unilateral, Loftus and Nicholls, 2012; Benwell et al., 2015). Thus, the cortical activation level at stimulation onset may have affected our results (Loftus and Nicholls, 2012); for instance, it has been shown that increased cognitive load (i.e., applying non-invasive brain stimulation during an ongoing task vs. no task) can increase the impact of the stimulation itself (Cappelletti et al., 2013). Hence, it may be easier to counteract an existing interhemispheric asymmetry than to exacerbate it. Though a bilateral electrode montage is particularly suitable to shift the interhemispheric balance, it does not show the isolated effects of anodal or cathodal stimulation (e.g., whether one hemisphere in particular drives the observed effects; Sehm et al., 2012; Benwell et al., 2015). Recent evidence indicates that shifts in spatial attentional biases might be driven by right-hemispheric alpha band power in the ventral attentional network (Benwell et al., 2017). This network controls non-spatial attentional aspects (e.g., alertness), is strongly lateralized toward the right hemisphere, and is assumed to closely interact with the dorsal attentional network (e.g., Corbetta and Shulman, 2002, 2011). Due to the relatively low spatial resolution of tDCS (Nitsche et al., 2008), we cannot exclude the possibility of a co-stimulation of adjacent nodes of the ventral attentional network (see also, e.g., Chechlacz et al., 2018). In fact, a hypothetical co-stimulation of areas of the ventral attentional network could potentially also explain the observed overall increase in RTs in the AL/CR condition (i.e., being observable only when inhibitory, cathodal stimulation takes place over the right hemisphere, due to the strong right-hemispheric lateralization of the ventral network, and affecting both the left and the right space, due to the non-spatial character of the latter). However, it has also been shown that the dorsal attentional network affects activity in the ventral attentional network independent of stimulation (e.g., Corbetta et al., 2008; DiQuattro and Geng, 2011; Vossel et al., 2014). Hence, a change in the excitability of the right PPC could potentially also have affected activity in the right ventral attentional network and ultimately resulted in increased overall RTs. Nevertheless, we did not find any differences in the subjective level of alertness (a non-spatial attentional aspect, governed by the ventral attentional network) between pre- and post-stimulation nor between the stimulation conditions, as revealed by the VAS. Another, non-mutually exclusive interpretation of the findings concerning the observed overall increase in RTs in the AL/CR condition is represented by different roles played by the two hemispheres: Whereas the left hemisphere would preferentially subtend attentional processing within the right, contralateral space, the right hemisphere would subtend attentional processing within both the left, contralateral space and the right, ipsilateral space (Heilman and Van Den Abell, 1980; Silver and Kastner, 2009; Sheremata and Silver, 2015). According to this account, the inhibitory, cathodal stimulation over the right hemisphere in the AL/CR condition would trigger detrimental effects on RTs in both the left and the right side of space, due to the aforementioned bilateral representation within the right hemisphere, thus potentially explaining the observed overall increase in RTs in this condition.

In conclusion, our findings provide, for the first time, evidence that cognitive load-related asymmetries in visuospatial attentional deployment are influenced by interhemispheric balance mechanisms between the left and right PPCs, which can be modulated in a polarity-specific fashion by means of tDCS.

## Data Availability Statement

The data supporting the conclusions of this study are available, in anonymized form, as Supplementary Material (see Supplementary Tables S1–S3).

## Ethics Statement

The study involving human participants was reviewed and approved by the Ethics committee of the University of Bern. The participants provided their written informed consent to participate in this study.

## Author Contributions

MB, UM, ThN, ToN, RM, and DC conceptualized and designed the experiments. LN programmed the experiments. RP, FW, and DC collected and analyzed the data. RP, MB, RM, and DC interpreted the results. RP and DC wrote the manuscript. All authors revised and approved the final version of the manuscript.

## Conflict of Interest

The authors declare that the research was conducted in the absence of any commercial or financial relationships that could be construed as a potential conflict of interest.
